# Effect of Generational Differences on the Quality of Anaesthesia Care: A Narrative Review

**DOI:** 10.7759/cureus.111525

**Published:** 2026-06-25

**Authors:** Anusha Balasubramanian, Sahanapanneer Selvam

**Affiliations:** 1 Anaesthesiology, Shri Sathya Sai Medical College and Research Institute, Chennai, IND

**Keywords:** anaesthesia services, generation gap, intergenerational communication, patient safety, workforce diversity

## Abstract

The healthcare workforce increasingly comprises multiple generations with differing values, communication styles, approaches to clinical decision-making, and levels of technology adoption. In anaesthesia, a speciality that relies on effective teamwork, rapid decision-making, and patient safety, these differences may influence the quality of care delivered. This narrative review examines how generational diversity among anaesthesia providers affects key domains of practice, including communication, clinical decision-making, technology adoption, patient safety, and work-related attitudes such as burnout and work-life balance. Senior anaesthesiologists contribute extensive clinical experience, crisis-management skills, and contextual judgment, whereas early-career clinicians often demonstrate stronger engagement with evidence-based practice, clinical guidelines, and digital technologies. While differences in communication styles, perceptions of hierarchy, and approaches to patient management may create challenges in perioperative care, they also provide opportunities for complementary learning and collaboration. Strategies such as structured communication, intergenerational mentorship, simulation-based training, and continuing professional development may help integrate the strengths of clinicians across career stages. Recognising and effectively managing generational differences can enhance teamwork, support patient safety, and improve the overall quality of anaesthesia services.

## Introduction and background

A significant demographic shift is underway across healthcare systems worldwide, with individuals from three or more generations simultaneously employed in the same clinical space (e.g., Baby Boomers, Generation X, Millennials, and now Generation Z) [1]. Each of the four generational cohorts has been shaped by distinct socio-cultural, educational, and technological experiences during their formative years [2]. Although generational characteristics have been described in the literature, their expression may vary across geographic regions because of differences in healthcare systems, cultural norms, workforce structures, and training environments [3]. Studies from North America, Europe, and other healthcare settings have nevertheless reported generational variation in attitudes toward authority, communication, learning preferences, and adaptability to change, all of which may influence professional interactions and clinical practice [3,4].

An anaesthetist's speciality is a high-risk, highly precise field that requires the anaesthetist to remain continuously aware of what is going on around them, make quick decisions, and coordinate with the surgeon, nurses, and all other staff caring for the patient preoperatively, intraoperatively, and postoperatively. A single lapse in communication or judgment can have negative consequences, regardless of how small it may be [5]. Therefore, the presence of generational differences within an anaesthesia team may be of great importance when considering the safety of the patients they care for, the efficiency with which anaesthesia care is delivered, and the overall quality of the anaesthesia services they provide [6]. Differences in professional training, clinical experience, exposure to evolving evidence, and familiarity with digital technologies may influence how anaesthesiologists approach patient care, communication, and clinical decision-making [7,8]. While some clinicians may draw more heavily upon experiential knowledge and intuitive judgment developed through years of practice, others may place greater emphasis on contemporary evidence, standardised protocols, and digital decision-support tools. These approaches are not mutually exclusive and often complement one another in modern anaesthesia practice.

While the aforementioned characteristics can yield synergistic benefits, they may also lead to communication breakdowns, differences in clinical priorities, closed-mindedness to change, and inconsistent adherence to evolving guidelines. Such inconsistencies are critical in high-stress environments such as the operating room, as they may negatively affect team dynamics and coordination among operating room personnel, thereby adversely affecting patient care. Although generational diversity among all healthcare employees has been increasingly identified, the majority of existing literature on this phenomenon tends to focus primarily on general healthcare environments, organisational behaviour, and nursing practice. There is minimal published, speciality-specific evidence in anaesthesia regarding the impact of generational differences on anaesthesia service delivery [9,10].

The significance of anaesthesiology in perioperative management and patient safety increases as we investigate how generational differences may lead to inconsistencies in the level of services provided [11,12]. Thus, this narrative review evaluates how generational differences among anaesthesiologists may influence key domains of anaesthesia practice, including communication and team dynamics, clinical decision-making, technology adoption, patient safety and risk management, and burnout/work-life balance. In addition, the review explores strategies to bridge generational differences and promote high-quality, safe anaesthesia care. These domains provide the framework through which the potential impact of generational diversity on anaesthesia service delivery is examined.

## Review

This narrative review explores the influence of generational differences on anaesthesia practice and service quality. Relevant literature was identified through searches of PubMed, Scopus, Google Scholar, and Web of Science using combinations of the terms “generation gap”, “generational differences”, “multigenerational workforce”, “anaesthesia”, “anesthesiology”, “communication”, “clinical decision-making”, “technology adoption”, “patient safety”, “risk management”, “burnout”, and “work-life balance”. Preference was given to peer-reviewed and anaesthesia-specific publications; however, relevant evidence from broader healthcare, patient safety, and organisational behaviour literature was also considered when speciality-specific studies were limited. The identified literature was reviewed and synthesised narratively across the major thematic domains examined in this review, including communication and team dynamics, clinical decision-making, technology adoption, patient safety and risk management, and burnout and work-life balance. Given the narrative nature of this review, formal inclusion and exclusion criteria, risk-of-bias assessment, and quantitative synthesis were not undertaken. The databases searched, key search terms, and search period used for this narrative review are summarised in Table 1.

**Table 1:** Literature search strategy used for this narrative review

Database	Search Terms (Examples)	Time Period Searched	Purpose
PubMed	"generation gap", "generational differences", "multigenerational workforce", "anaesthesia", "anesthesiology", "communication", "clinical decision-making", "technology adoption", "patient safety", "burnout", "work-life balance"	January 2000 - April 2026	Identification of peer-reviewed healthcare and anaesthesia literature
Scopus	Same search strategy with database-specific adaptations	January 2000 - April 2026	Broad multidisciplinary literature search
Web of Science	Same search strategy with database-specific adaptations	January 2000 - April 2026	Citation-based and multidisciplinary literature search
Google Scholar	Combination of key search terms and manual reference screening	January 2000 - April 2026	Identification of additional relevant publications and grey literature

Communication and team dynamics

Communication is paramount to effective clinical anaesthesia practice; during the perioperative period (before, during, and after surgery), when patient safety can be greatly compromised, patient information must be shared among members of the surgical team in an appropriate and timely manner through appropriate communication channels [13].

Generational differences among anaesthesia providers can create significant barriers to effective communication among team members [14]. Older generations in the anaesthesia community tend to communicate in a very structured, hierarchical manner, as a result of the tradition-based training they received; they rely on authority-based communication models and a well-defined chain of command to transmit and receive information [15]. On the other hand, younger providers (e.g., Millennials/Generation Z) tend to favour more collaborative, inclusive, and technology-based forms of communication (e.g., digital platforms, immediate access to data) when communicating with one another and their surgeons [16]. These different styles of communication can create barriers to effective communication and lead to misunderstandings, where the intent of the transmitted information is misinterpreted. Anxious junior practitioners may be reluctant to question the authority of their more senior counterparts, and a lack of cohesion within a team can negatively affect communication and team performance during high-risk situations. To achieve high performance from an operating room surgical team, there must be smooth coordination among anaesthesiologists, surgeons, and nurses, as well as effective communication among team members. It is well known that poor communication has been implicated in many errors that occur perioperatively, as well as in many adverse outcomes [10]. Generational diversity can improve communication within teams when properly managed. Older clinical professionals are often able to demonstrate clarity of intent, decisiveness, and leadership, while younger clinical professionals can demonstrate flexibility and inclusion. Developing structured methods of communication (for example, using checklists, conducting preoperative briefings, and performing postoperative debriefings) can help reduce generational barriers to effective communication and work toward standardising communication across team members in a surgical setting. In addition to removing communication barriers to improve patient safety, it will also be necessary to develop and maintain a culture where all team members feel psychologically safe [5,17,18]. Table 2 shows characteristics of different generations in healthcare [4,19].

**Table 2:** Characteristics of different generations in healthcare Generational cohorts are demographic categories and should not be considered equivalent to professional rank. In anaesthesia practice, clinicians from different generations may occupy a range of professional roles, including consultant/attending anaesthesiologists, fellows, residents, and trainees. Created by the authors based on information reported in [4,19].

Generation	Birth Years	Key Traits	Communication Style	Approach to Work	Technology Adaptation
Baby Boomers	1946-1964	Experienced, disciplined	Hierarchical, formal	Work-centric	Moderate
Generation X	1965-1980	Independent, adaptable	Direct, pragmatic	Balanced	Good
Millennials	1981-1996	Team-oriented, flexible	Collaborative	Work-life balance	High
Generation Z	>1996	Tech-savvy, fast learners	Digital, instant	Flexible	Very high

Clinical decision-making

Many factors influence an anaesthetist's decision-making, including clinical knowledge, situational awareness, prior experience, individual risk perception, and the clinical context in which care is delivered. While generational differences may influence attitudes toward evidence-based practice, technology use, and information access, clinical decision-making in anaesthesia is primarily shaped by professional training and clinical experience. Studies examining decision-making under uncertainty have demonstrated variation in how clinicians interpret risk, process information, and select management strategies; however, these differences cannot be attributed solely to generational membership [20].

For example, during unexpected intraoperative events such as difficult airway management, malignant hyperthermia, or sudden haemodynamic instability, clinicians may combine evidence-based recommendations, crisis-management protocols, team communication, and clinical judgment when determining an appropriate course of action. Individual approaches may differ according to training background, familiarity with guidelines, and previous exposure to similar situations. Consequently, decision-making in anaesthesia should be viewed as a dynamic process that integrates both experiential knowledge and contemporary evidence rather than as a characteristic of a particular generation [20,21].

The increasing emphasis on evidence-based practice has promoted the use of standardised protocols and decision-support systems, helping to reduce variability in care and improve adherence to accepted standards of practice. However, strict adherence to guidelines alone may not always address the complexity of individual patients, particularly those presenting with rare conditions, multiple comorbidities, or atypical clinical circumstances. In such situations, clinical experience and flexibility remain important components of safe and effective anaesthetic care [21].

Differences in clinical decision-making approaches may occasionally lead to disagreement within anaesthesia teams, particularly when clinicians have varying levels of experience, training backgrounds, or familiarity with evolving evidence. For example, differing opinions may arise regarding the application of clinical guidelines, the interpretation of risk, or the choice between standardised and individualised management strategies for complex patients. When these differing perspectives are not discussed constructively, they may contribute to communication challenges, interpersonal tension, or delays in decision-making. Conversely, open discussion and collaborative decision-making can enable clinicians to integrate multiple perspectives and arrive at more balanced patient-management decisions [21,22].

Despite these differences, experiential knowledge and evidence-based practice should be viewed as complementary rather than competing approaches. Combining the clinical judgment of experienced anaesthesiologists with the contemporary knowledge and guideline familiarity of younger clinicians can enhance decision-making, improve patient safety, and reduce the likelihood of errors [23]. Encouraging collaborative discussion and participatory decision-making allows clinicians across career stages to contribute their respective strengths, fostering a culture of shared learning and supporting the delivery of high-quality anaesthesia care [24]. The impact of generational differences on anaesthesia practice is shown in Table 3 [25].

**Table 3:** Impact of generational differences on anaesthesia practice Created by the authors based on information reported in [25].

Domain	Older Generations	Younger Generations	Impact on Quality
Clinical decision-making	Experience-based	Evidence-based	Complementary but may conflict
Communication	Hierarchical	Collaborative	Risk of miscommunication
Technology use	Slower adoption	Rapid adaptation	Variation in practice
Patient safety	Experience-driven	Protocol-driven	Improved if integrated
Work style	Long hours	Balanced lifestyle	Risk of burnout vs efficiency

Adoption of technology

Technological innovation has transformed modern anaesthetic practice through the introduction of electronic health records, anaesthesia information management systems, advanced haemodynamic monitoring, depth-of-anaesthesia monitoring, ultrasound-guided regional anaesthesia, decision-support systems, telemedicine platforms, and simulation-based medical education [26]. These technologies have improved perioperative monitoring, documentation accuracy, clinical decision-making, and training opportunities, thereby contributing to enhanced patient safety and efficiency.

Differences in technology adoption may be influenced by training background, exposure to digital tools, institutional resources, and individual preferences. Clinicians who trained in more digitally integrated environments may demonstrate greater familiarity with technologies such as electronic anaesthesia records, point-of-care ultrasound, and digital decision-support tools. However, technology adoption should not be viewed solely through a generational lens, as many experienced consultant and attending anaesthesiologists have successfully integrated advanced monitoring systems, electronic documentation platforms, and simulation-based education into their clinical practice. Variability in technology use may therefore reflect differences in training experiences and workplace environments rather than age alone [27,28].

The integration of technology into anaesthesia practice has been associated with earlier recognition of physiological deterioration through advanced haemodynamic monitoring, improved documentation through electronic anaesthesia records, and increased procedural safety through ultrasound-guided regional anaesthesia. Nevertheless, excessive reliance on technology may create additional challenges by reducing clinical vigilance and diminishing reliance on fundamental clinical assessment skills [29]. Consequently, the optimal use of technology requires a balance between technological innovation and sound clinical judgment [30].

Ongoing educational initiatives that combine formal instruction with hands-on experience are essential for promoting the effective use of emerging technologies across clinicians at different career stages. Such programs can facilitate knowledge exchange, encourage technology adoption where appropriate, and support the integration of innovative tools into anaesthesia practice while maintaining high standards of patient care [31].

Patient safety and risk management

The primary goal of anaesthesia practice is to ensure patient safety, and the outcome depends heavily on maintaining vigilance, following processes, and having effective strategies for managing crises [32]. Generational differences can create variation in how clinicians assess risk and apply safe practices. Senior clinicians have been able to manage crisis situations, as well as deal with intricate and infrequent complications overall. This expertise, which allows for the ability to detect variations in a person's clinical status over time, helps facilitate timely patient intervention [33].

Conversely, junior clinicians tend to follow safety protocols, checklists, and evidence-based clinical guidelines more stringently than their senior counterparts. The use of the WHO Surgical Safety Checklist [34] has increased patient safety in perioperative environments by creating systematic methods of procedure-based communication and confirming systems prior to commencing the procedure.

Differences in training experiences and professional socialisation may influence approaches to patient safety and risk management. Studies examining safety culture in perioperative and critical care environments have demonstrated that factors such as communication practices, teamwork, situational awareness, and adherence to safety processes can vary among healthcare professionals with different training backgrounds and levels of clinical experience [5,10,17,18]. Regardless of generation, clinicians generally share the common goal of maintaining patient safety through vigilance, effective communication, and adherence to recognised standards of care. Variability may therefore reflect differences in training, workplace culture, and professional experience rather than generational characteristics alone.

Although both approaches contribute positively to patient safety, differences in risk perception and clinical decision-making strategies may occasionally influence patient management [35]. Experienced clinicians may adapt care based on clinical judgment and prior experience, whereas junior clinicians may place greater emphasis on protocol-driven practice. When integrated through effective teamwork, structured communication, simulation-based training, and standardised safety processes, these complementary perspectives can strengthen patient safety and promote consistent delivery of high-quality anaesthesia care [10,23,34].

Burnout and work-life balance

**Figure 1:**
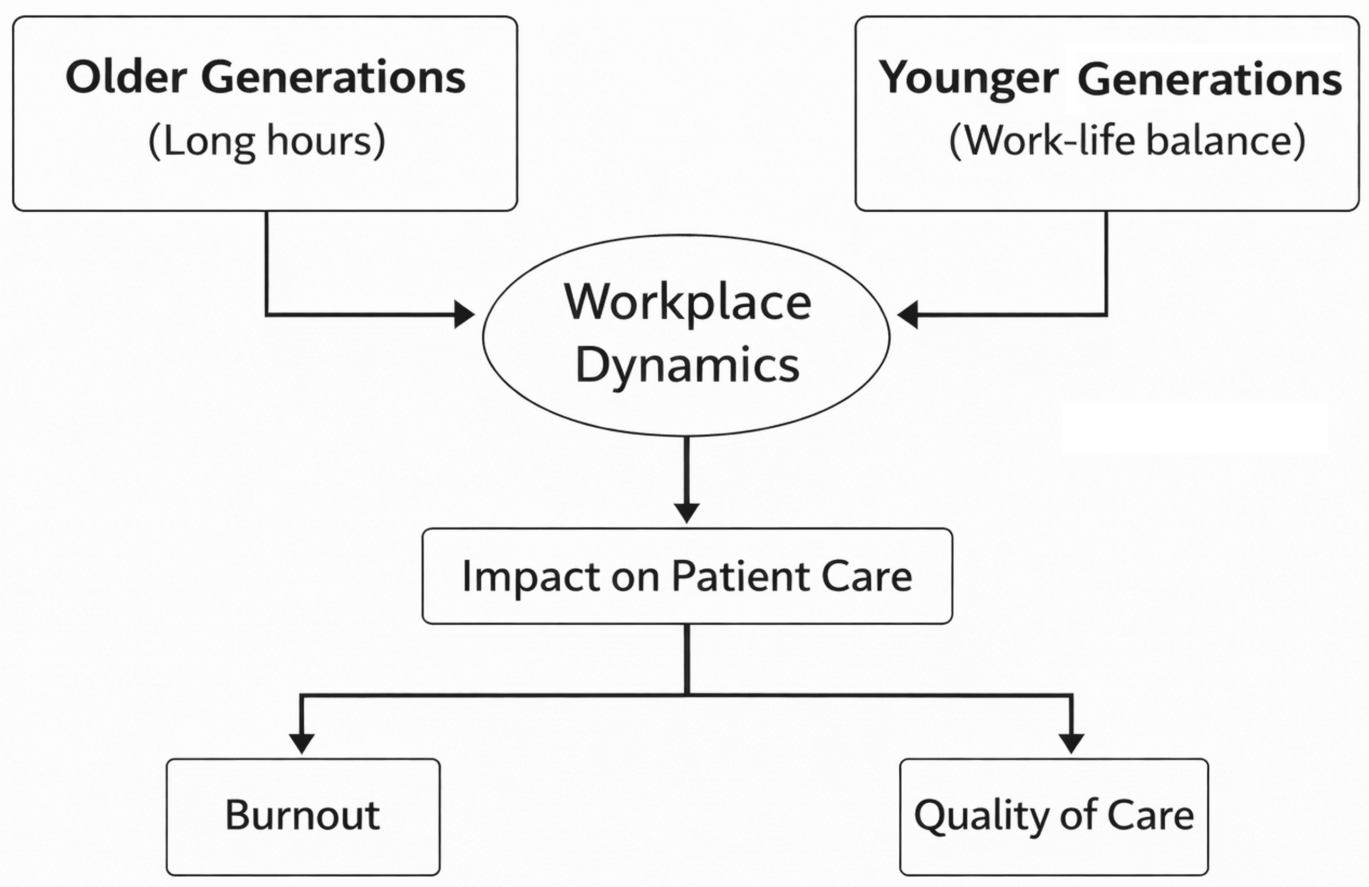
Conceptual framework of generational differences in work attitudes and their impact on patient care Created by the authors using Microsoft PowerPoint (Microsoft Corp., Redmond, USA).

Generational differences in approaches to professional life and work practices may influence job satisfaction, burnout, and ultimately the quality of patient care [36]. Older generations, particularly Baby Boomers, were often trained in healthcare environments where extended working hours and high levels of professional dedication were common. While these work patterns contributed to the development of extensive clinical experience and expertise, prolonged exposure to demanding schedules may increase the risk of fatigue, burnout, and reduced well-being among clinicians [34,37]. Figure 1 illustrates how differing perspectives on work practices, occupational well-being, and work-life integration may influence workplace interactions, team dynamics, and patient care outcomes, including clinician burnout and quality of care.

In contrast, Millennials and Generation Z clinicians generally place greater emphasis on occupational health, work-life integration, mental well-being, and flexible working environments [38]. Growing awareness of the effects of burnout on healthcare professionals and patient outcomes has encouraged many clinicians to prioritise self-care, workload management, and sustainable career development. Although these evolving priorities may sometimes be perceived differently across generations, they largely reflect changing perspectives on workforce well-being rather than differences in professional commitment to patient care [39,40].

Burnout has been linked to an increase in medical errors, lower patient satisfaction and a decrease in safety for anaesthesiologists. Therefore, being aware of the differences between the two groups, institutions must find ways to address generational differences in work ethics to provide excellent patient care. Institutions should work to foster a supportive environment, ensure open communication with employees about their workload and overall well-being, and develop policies and practices that support both productivity and mental health [40]. An environment that creates mutual respect for generational work habits amongst employees may produce a more cohesive and resilient workforce [41].

Recommendations for bridging generational differences in anaesthesia practice

When developing strategies to effectively mitigate the generational differences that affect anaesthetic practice, introducing targeted interventions will facilitate collaboration and leverage the complementary strengths of both generations. The first intervention is to implement structured communication tools, such as preoperative briefings, intraoperative checklists, and postoperative briefings, that would enable standardised communication between anaesthetists from each generation regarding their patients' care, thereby increasing the likelihood of accurate and timely information exchange about their patients' anaesthetics. In addition, implementing routine simulation training programs would provide both generational cohorts with an opportunity to disseminate clinical knowledge through mentorship from 'senior' anaesthetists to these newer anaesthetists and share their own evidence-based clinical practices and technology [42]. Both generations will contribute to the learning and development process by providing 'bi-directional' mentoring to each other. Lastly, continuing medical education (CME) courses and lectures for anaesthetists need to include a variety of teaching methods, both traditional and technology-based, that address generational learning differences. Finally, opportunities for intergenerational competence can also be developed through participation in workshops that provide educational and experiential tools for effective communication, adaptability, and collaboration [43].

At the policy level, healthcare organisations must build a culture of psychological safety that fosters an environment where every team member can freely voice their concerns without regard to rank or title [44]. Policies that are flexible and consider fair distributions of workload and effort are particularly important for maintaining the health and productivity of the workforce within healthcare organisations [45]. The training programs for multigenerational healthcare leaders should focus on developing progressive management practices that include and acknowledge the various generations represented within healthcare organisations [46,47].

In conclusion, adopting a structured, team-based model of anaesthesia practice, along with values of experience and innovation, is key to maximising anaesthesia service delivery quality within a multigenerational workforce [48]. The characteristics and major findings of the most relevant studies included in this narrative review are summarised in Table 4.

**Table 4:** Summary of key studies included in this narrative review

Author (Year)	Study Type	Population/Setting	Key Findings Relevant to Generational Differences
Burton-Anderson et al. (2026) [1]	Research report	Healthcare workforce	Generational differences influence communication, teamwork, leadership, and workplace expectations.
Ravid et al. (2025) [3]	Systematic review	Multigenerational workforce	Evidence for generational differences is mixed; workplace factors often contribute more strongly than age cohort alone.
Cecconi et al. (2025) [4]	Review article	Healthcare workforce	Technology adoption varies across generations, although adaptation is influenced by training and organisational support.
Reader et al. (2006) [5]	Review article	Critical care and perioperative environments	Communication, teamwork, and non-technical skills are essential for patient safety.
Sexton et al. (2006) [10]	Cross-sectional survey	Operating room personnel	Differences in teamwork perceptions and safety culture may affect perioperative performance.
Radhakrishnan et al. (2022) [17]	Review article	Anaesthesia education	Non-technical skills and teamwork contribute significantly to safe anaesthesia practice.
Matos et al. (2020) [18]	Longitudinal observational study	Anaesthesiology residents	Development of non-technical skills improves with training and clinical exposure.

Strengths and limitations

This review offers a focused synthesis of an underexplored yet clinically relevant aspect of anaesthesia practice by specifically examining generational dynamics within a high-stakes, team-dependent speciality. It integrates perspectives from organisational behaviour, patient safety, and clinical decision-making to construct a multidimensional understanding of how generational diversity may influence perioperative care. The thematic structuring across domains such as communication, technology use, and risk management enhances conceptual clarity and practical applicability for anaesthesia teams. However, certain limitations must be acknowledged. The interpretive nature of a narrative review introduces the possibility of selective inclusion and subjective emphasis, potentially affecting the neutrality of conclusions. Additionally, the reliance on extrapolated evidence from broader healthcare settings, due to limited anaesthesia-specific studies, may restrict contextual precision. Variability in defining generational cohorts across studies further complicates direct comparisons. Finally, the absence of quantitative synthesis precludes objective estimation of effect size or causality. Furthermore, because many observations regarding generational differences overlap with factors such as clinical experience, career stage, and workplace culture, distinguishing the independent effect of generation remains challenging. These limitations highlight the need for future empirical and anaesthesia-specific research.

## Conclusions

Generational diversity within the anaesthesia workforce presents both challenges and opportunities. Senior anaesthetists contribute extensive clinical experience and crisis management skills, while younger generations enhance practice through technological proficiency, adaptability, and evidence-based approaches. Although differences in communication styles, hierarchy perception, and clinical decision-making may affect teamwork and perioperative coordination, these barriers can be addressed through structured communication strategies, intergenerational mentorship, continuous professional development, and supportive workplace policies. Effectively integrating the strengths of all generations can improve teamwork, innovation, patient safety, and the overall quality of anaesthesia services.
